# MiR-124 and miR-506 are involved in the decline of protein C in children with extra-hepatic portal vein obstruction

**DOI:** 10.1038/s41598-021-91862-4

**Published:** 2021-06-10

**Authors:** Jin-Shan Zhang, Long Li

**Affiliations:** Department of General Surgery, Capital Institute of Pediatrics, No. 2 Yabaolu Rd., Beijing, 100020 People’s Republic of China

**Keywords:** Upper gastrointestinal bleeding, Non-coding RNAs, Disease genetics, Genetics research, Paediatric research

## Abstract

The deficiency of protein C (PROC) can be partly rescued by Rex shunt through restoring portal blood flow in children with extra-hepatic portal venous obstruction (EHPVO). However, the decline of PROC is still found in some patients with a normal portal blood flow after Rex shunt. The aim of this study was to identify the candidate miRNAs involving in the decline of PROC and their mechanism. The protein level of PROC was detected by the ELISA assay, and was compared between sick and healthy groups. The expressions of miRNAs and PROC mRNA were measured using qRT-PCR, and were compared between sick and healthy groups. The correlation between PROC and candidate miRNAs was analysed by a Pearson correlation analysis to identify the most significant miRNAs. The expression of PROC mRNA was detected by qRT-PCR in HL-7702 and LX-2 cells tansfected with miRNAs mimics or inhibitors and negative control (NC) mimics, which was compared among the different groups. The rates of liver cells’ proliferation and apoptosis were detected in HL-7702 and LX-2 cells tansfected with miRNAs mimics or inhibitors or with overexpressing PROC and negative control mimics by CKK8 assay and flow cytometry, which were compared among the different groups. The expressions of COX-2 and VEGF were measured by qRT-PCR, and were compared between the miRNAs groups and NC group. Western blot was assayed for detecting the protein levels of PROC, COX-2, VEGF, Bcl-2 and Bax, which were compared between the miRNAs groups and NC group. The expression of PROC mRNA was lower, and the expressions of miR-506-3p and miR-124-3p were higher in children with EHPVO than healthy group. PROC mRNA was negatively correlated with the expression of miR-506-3p and miR-124-3p. Compared to the NC group, the transcription activity of PROC was lower after exposure of miR-506 and miR-124 mimics in HL-7702 and LX-2 cells, but this phenomenon was reversed after inhibiting miR-506 and miR-124. The rate of cell proliferation was lower after exposure of miR-506 and miR-124 than the NC group, which was increased after inhibiting miR-506 and miR-124 in HL-7702 cells and overexpressing PROC in LX-2 cells. The apoptotic rate was higher after exposure of miR-506 and miR-124 than the NC group, which was decreased after inhibiting miR-506 and miR-124 in HL-7702 cells and overexpressing PROC in LX-2 cells. The mRNA levels of COX-2 and VEGF were significantly higher after exposure of miR-506 and miR-124 mimics than those in the NC group. The protein levels of PROC and Bcl-2 were down-regulated, and the levels of COX-2, Bax and VEGF were up-regulated after exposure of miR-506 and miR-124 in HL-7702 cells, but this phenomenon was reversed after inhibiting miR-506 and miR-124. MiR-506-3p and miR-124-3p may involve in the decline of PROC in protein and transcriptional level, in which the anti-proliferation and pro-apoptosis role of miR-506-3p and miR-124-3p for liver cells may involve in this mechanism.

## Introduction

Protein C (PROC) is synthesized in liver, and exists potential anticoagulant activity^1^. The functional PROC deficiency is considered to be one of the primary causes of deep vein thrombosis (DVT)^2^. More than 31% of the children with extra-hepatic portal venous obstruction (EHPVO) have functional PROC deficiency^3^, which may be caused by the reduced hepatopetal portal blood flow. Therefore, the deficiency of PROC can be partly rescued by Rex shunt through restoring portal blood flow^4^. However, the decline of PROC is still found in some patients with a normal hepatopetal portal blood flow after Rex shunt in our series, which suggests that the hepatopetal portal blood flow is not the only factor that affects the level of PROC.

The up-regulation of miR-4525, miR-451a and miR-21 levels in portal vein blood contributes to the development of pancreatic ductal adenocarcinoma^5^. And the down-regulation of miR-381 in portal vein tumor is closely associated with hepatocarcinogenesis^6^. Therefore, miRNAs may be involved in the progression of portal vein-related disease. In addition, two PROC nonsense mutations (p.Trp247* and p.Arg199*) trigger nonsense-mediated mRNA decay (NMD), resulting in PROC deficiency^7^. The transcriptional level of PROC is decreased after transfecting miR-24 and miR-34a, which may affect the development of thrombotic or hemorrhagic disorder^8^. Based on these findings, we suspected some miRNAs might involve in the decline of PROC in children with EHPVO. Therefore, this study is to identify the role and mechanism of miRNAs for the decline of PROC in children with EHPVO.

## Materials and methods

### Subjects

From December 2013 to June 2016, the blood samples from 53 children with EHPVO before and after Rex shunt and 18 normal children were collected, and the concentration and activity of serum PROC was detected by ELISA assay, which showed that the level of PROC in 6 children with EHPVO before and after Rex shunt were lower than that of the normal children. Postoperative ultrasound and computerized tomography (CT) showed that the bypass vein was patency and the blood flow into the liver was normal in this 6 patients. There was no postoperative upper gastrointestinal bleeding in the 6 patients. In addition, there was no family history of DVT in the 6 children, and their parents had normal level of PROC. Therefore, the 6 children with EHPVO were diagnosed as the decline of PROC. In this study, the blood samples of this 6 children after Rex shunt were enrolled as the sick group, and the blood samples of 6 normal children were used as the healthy group. This study was approved by the ethics committee of Capital Institute of Pediatrics. The informed consent was obtained from all participants’ parent.

### ELISA assay for the concentration of serum PROC

The concentration of serum PROC was detected in the sick and healthy groups by ELISA assay. Firstly, serum was acquired after centrifugation at 9000 rpm for 3 min at 4 °C. Then detection of serum PROC were carried out according to ELISA kit instructions. The OD value was detected at the wavelength of 560 nm in microplate reader (Thermo Fisher, Multiskan MK3) and the concentration of serum PROC was calculated by the standard curve.

### Real-time PCR amplification

Total RNA was extracted from the samples (blood or liver samples) using Trizol reagent according to the instructions of the manufacturer. mRNA was reverse transcribed using Oligo (dT) or miRNA-specific inverse primers through the instructions of the manufacturer^9^. PCR amplification were performed using the All-in-One miRNA qRT-PCR Detection Kit (GeneCopoeia) or iTaq SYBR green Master Mix kit (Vazyme, q111-02). Real-time PCR analyses were carried out using CFX96 instrument (Bio-Rad). Primers sequence were showed in Table 1. RT-PCR expression data were normalized to the selected reference gene using GAPDH. The data analysis of qRT-PCR is performed by the 2^−△△Ct^ method.

**Table 1:** Primer sequences for qRT-PCR.

Gene	Sense (forward primer)	Antisense (reverse primer)
U6	CTCGCTTCGGCAGCACATATACT	ACGCTTCACGAATTTGCGTGTC
miR-204-5p	CGTTCCCTTTGTCATCCT	GTGCAGGGTCCGAGGT
miR-211-5p	CGTTCCCTTTGTCATCCT	GTGCAGGGTCCGAGGT
miR-218-5p	CGCGTTGTGCTTGATCTAA	GTGCAGGGTCCGAGGT
miR-802	CGCAGTAACAAAGATTCAT	GTGCAGGGTCCGAGGT
miR-124-3p	CGTAAGGCACGCGGTG	GTGCAGGGTCCGAGGT
miR-506-3p	CGTAAGGCACCCTTCTG	GTGCAGGGTCCGAGGT
PROC	5′-CTGCACGCATTACTGCCTAGA-3′	5′-CCTCCCACAAGGGAACTTCA-3′
GAPDH	5′-TGGTATGACAACGAATTTGG-3′	5′-TCTACATGGCAACTGTGAGG-3′

### Luciferase assay

Firstly, the wild-type (WT) or mutant-type (MT) 3′-UTR sequence of PROC (WT: TCACATGCCTTAT; MT: TGTGTCAGGCCGA) were amplified from the genomic DNA and sub-cloned into the region located at downstream of the stop codon in the luciferase gene in the luciferase reporter vector (pGL4-Basic system)^10^. Then, the reporter plasmid and miRNA mimics or Renilla were co-transfected into 293 T cells by Lipofectamine 2000 (Invitrogen, USA). The 293 T cells were cultured in complete medium containing 10% FBS at 37 °C with 5% CO_2_ for 48 h. At last, the luciferase activity were measured using the dual-luciferase reporter assay system (Promega, USA) in the different transfected cells.

### Cell transfection

To identify the role of miRNA on the decline of PROC in different cell lines, HL-7702 and LX-2 cells were used to repeat this study. HL-7702 and LX-2 cells were cultured in RPMI complete medium containing 10% fetal bovine serum (FBS) at 37 °C with 5% CO_2_. Cells were plated at a density of 3 × 10^5^ in 6-well plates before cell transfection. After 12 h of cultivation, cells transfection was performed according to the transfection steps of the Lipofectamine 2000 kit. 6 µl of lipofectamine 2000 (Invitrogen, 11,668–019) was incubated in 250 µl of Serum free 1640 as A and 50 ng miRNA mimics or their inhibitors was diluted with 250 µl of Serum free 1640 as B at room temperature for 5 min. The A mixture was then gently added into the B mixture at room temperature for 20 min. Then the final mixture was transferred into the 6-well plate that contained cells. After 4 h of incubation at 37 °C, the mixture was replaced with fresh media containing FBS albumin (10%). After 24 h of transfection, cells were harvested for the subsequent experiment. In this study, the cells transfected by miRNA mimics and their inhibitors were regarded as the miRNA group and miRNA inhibitors group, and the cells transfected by negative control (NC) mimics were regarded as the NC group. In addition, CDS fragments of PROC were sub-cloned into pEGFP-C1 plasmid, and this recombinant PROC-overexpressed plasmid were further transfected into cells by Turbofect transfection reagent (R0531, Thermo Fisher, USA).

### CCK8 assay

HL-7702 cells were transfected with miR-506 mimics, miR-506 inhibitor, miR-124 mimics, miR-506 inhibitor and negative control miRNA (NC miRNA). LX-2 cells were transfected with miR-506 mimics, miR-124 mimics, miR-506 with overexpressing PROC, miR-124 mimics with overexpressing PROC and negative control miRNA (NC miRNA). The above-mentioned cells were seeded at 96-well plate (5000 cells/well) and incubated for 24 h, 48 h, 72 h and 96 h. Following incubation, 20 µl CCK8 (Biosharp, BS350B) was added into each well, and the cells were cultured at 37 °C for additional 4 h. Microplate reader (Thermo Fisher, Multiskan MK3) was used to determine the absorption value of cells at the wavelength of 450 nm for measuring the viability of cells’ proliferation.

### Apoptosis assay

After transfected with miR-506 mimics/inhibitor, miR-124 mimics/inhibitor and NC miRNA for 24 h, apoptotic rate of HL-7702 cells was measured by flow cytometry^11^. LX-2 cells was transfected with miR-506 mimics or with overexpressing PROC and miR-124 mimics or with overexpressing PROC, and the apoptotic rate of LX-2 cells was measured by flow cytometry after 24 h transfection. In the flow cytometry, 5 µl Annexin V-FITC and 5 µl PI was added into each well and the cells were cultured at room temperature for 15 min after a gentle blending. Then, 400 µL PBS was added into each well and the cells were tested by streaming cell machine for measuring the apoptotic rate of cells.

### Western Blotting

Total protein was extracted from the transfected HL-7702 cells using RIPA buffer. The protein concentration was then quantified and normalized to ensure equal loading into polyacrylamide gels. Equal amounts of protein were subjected to 10% polyacrylamide gel electrophoresis and transferred to PVDF membrane. The membrane was incubated with the antibody of PROC (1:1000, ab180733, Abcam, UK), cyclooxygenase-2 (COX-2) (1:1000, ab179800, Abcam, UK), vascular endothelial-derived growth factor (VEGF) (1:2000, ab46154, Abcam, UK), B cell lymphoma-2 (Bcl-2) (1:1000, ab182858, Abcam, UK) and Bcl-2 associated X (BAX) (1:800, ab32503, Abcam, UK) overnight. The β-actin antibody (Cell Signaling Technology,USA) was served as the internal control. The next day, the membrane was incubated with the secondary antibody. The bands were visualized using an ECL luminescent reagent (Millipore, USA).

### Statistical analysis

Data was shown at mean ± standard deviation (SD). The statistical analysis was performed using the Statistical Package for the Social Sciences (SPSS) software (version: 25.0). The concentration of serum PROC and the expressions of miRNAs and PROC mRNA between the sick and healthy groups were compared using a Student’s t-test. The correlation between PROC mRNA and miRNAs was analysed by a Pearson correlation analysis. The level of PROC mRNA, the rates of proliferation and apoptosis, the transcriptionanl level of COX-2 and VEGF and the protein levels of PROC, COX-2, VEGF, Bcl-2 and Bax were compared between the miRNA and NC groups using a two-way analysis of variance (ANOVA). There was significant difference when *P* < 0.05.

### Ethics approval and consent to participate

This study was approved by the ethics committee of Capital Institute of Pediatrics. The informed consent was obtained from all participants’ parent.

### Consent for publication

The consent for publication have been obtained from all enrolled subjects.

### Statement

All methods used in this study were carried out in accordance with relevant guidelines and regulations.

## Results

### The miRNAs associate with the decline of PROC

**Figure 1:**
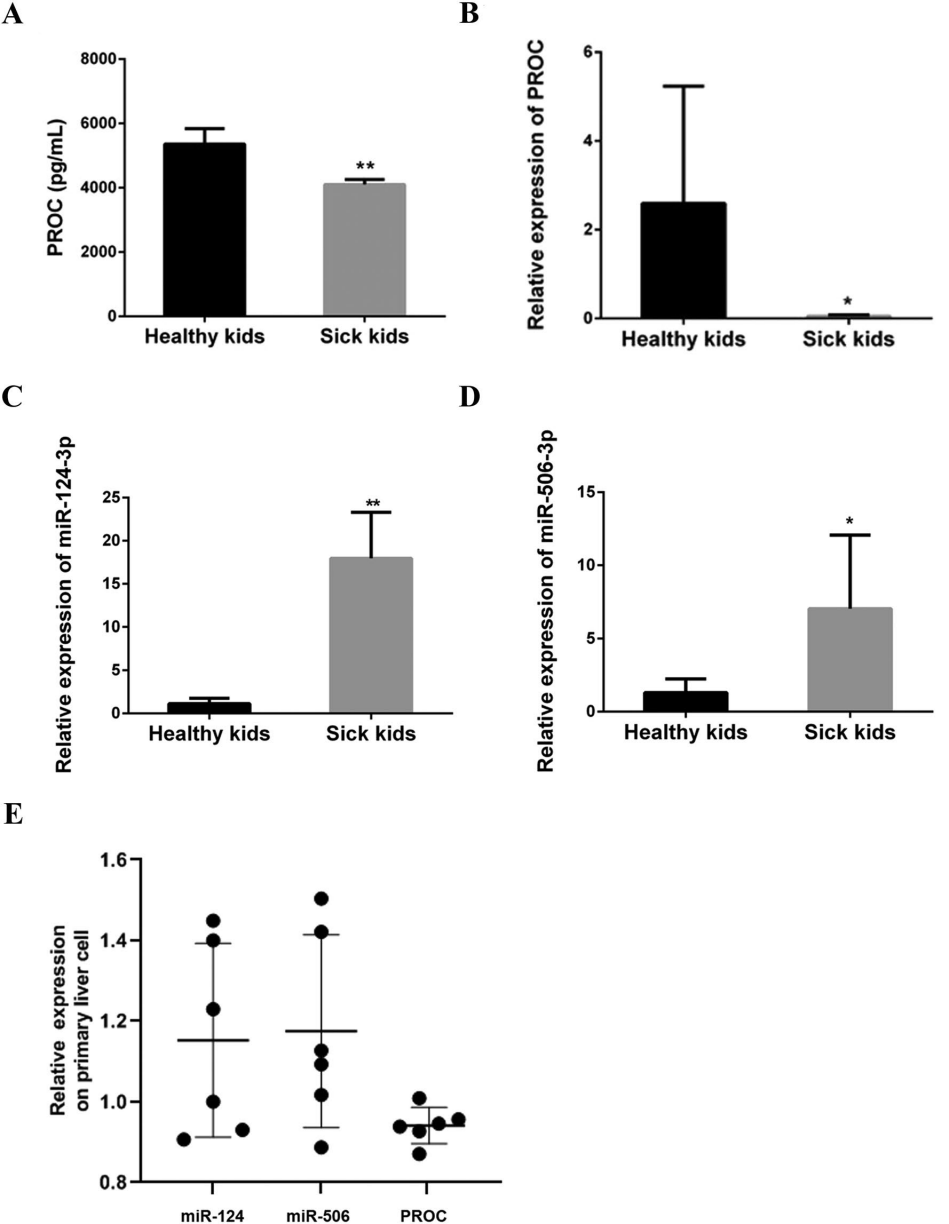
Detection of PROC and miRNAs. The protein and transcriptional levels of PROC was detected by ELISA (**A**) and qRT-PCR (**B**) in healthy kids and sick kids. The expressions of miR-124-3p (**C**) and miR-506-3p (**D**) were detected by qRT-PCR in healthy kids and sick kids. Healthy kids means whole blood samples of normal children. Sick kids means whole blood samples of children with EHPVO after Rex shunt. (**E**) Primary liver cells were harvested from mice live, and mRNA levels of PROC, miR-506 and miR-124 were determined by qRT-PCR in the primary liver cells. **Represents *P* < 0.01, *represents *P* < 0.05.

The protein and transcriptional levels of PROC were lower in sick kids than those in healthy kids (Fig. 1A, B, Supplementary Figs. 1–2). To forecast the miRNAs associated with PROC, the miRNA target gene prediction websites, including miRanda (http://www.microrna.org/) and TargetScan (http://www.targetscan.org/), were adopted. After integrating the predicted results, the most significant 6 miRNAs, including miR-204-5p, miR-211-5p, miR-218-5p, miR-802, miR-124-3p and miR-506-3p, were enrolled in this study. The levels of miR-204-5p, miR-211-5p, miR-218-5p and miR-802 were lower in sick kids than those in healthy kids, but there was no significant difference (Supplementary Figs. 3–6). The expressions of miR-124-3p and miR-506-3p in sick kids were significantly higher than those in healthy kids, while the expression of protein C mRNA in sick kids was significantly lower than that in healthy kids (Fig. 1B–D, Supplementary Figs. 7–8). There was a significant negative correlation between the expressions of miR-211-5p (r = − 0.656, *P* = 0.003), miR-802 (r = − 0.538, *P* = 0.021), miR-124-3p (r = − 0.334, *P* = 0.027) and miR-506-3p (r = − 0.840, *P* < 0.001) and the expression of PROC mRNA in sick kids. These results suggest that miR-124-3p and miR-506-3p may be associated with the decline of PROC.

Furthermore, the primary liver cells were harvested from 6 wild type mice, and the levels of PROC mRNA and miR-124/miR-506 were detected using qRT-PCR in each mice (Fig. 1E). There was a negative correlation between PROC and miR-124 (r = − 0.636, *P* = 0.034).

### miR-506 and miR-124 bind to the 3′UTR of PROC

**Figure 2:**
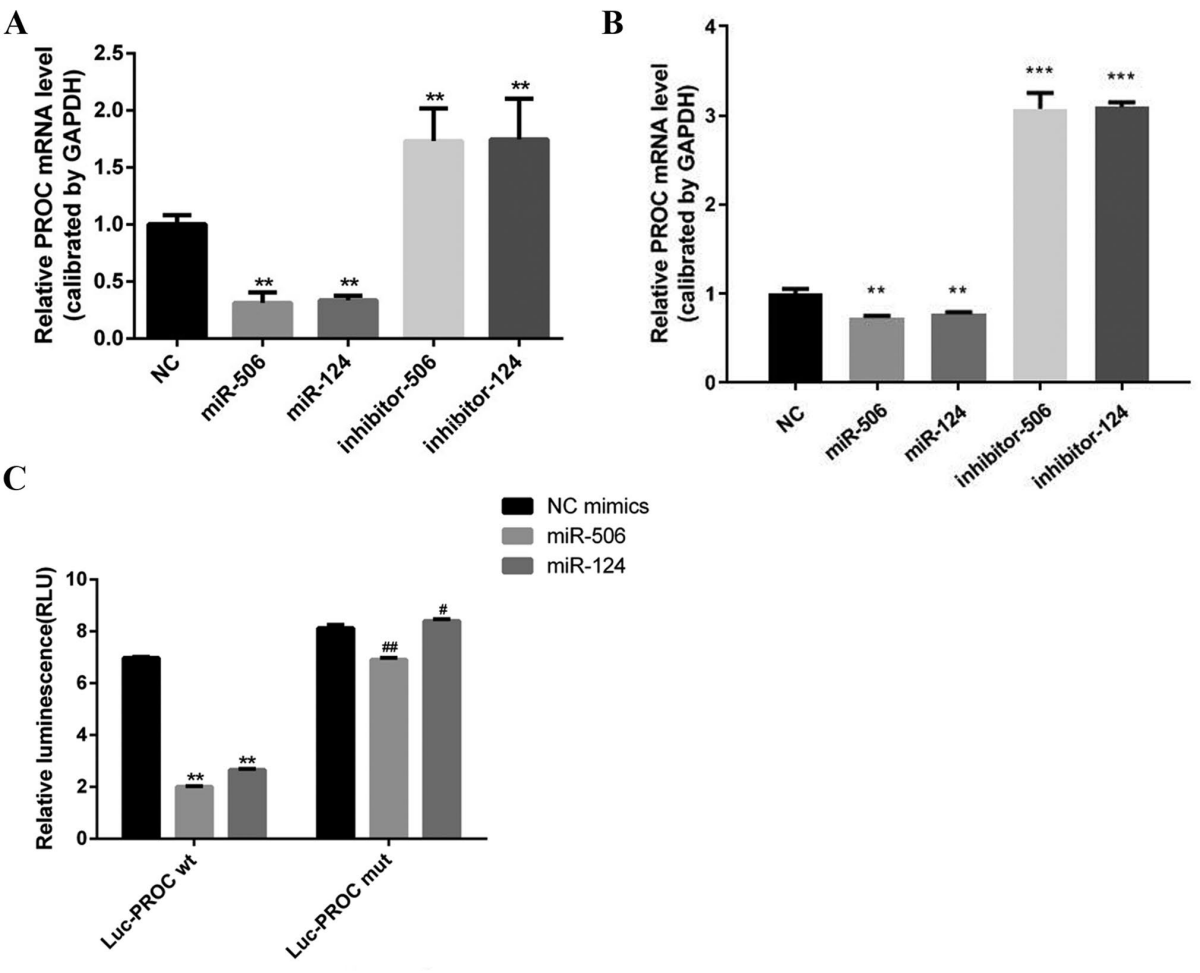
The association of miR-506/124 and PROC. Detection of PROC mRNA in HL-7702 cells (**A**) and LX-2 cells (**B**) transfected with miR-506 or miR-124 mimics, inhibitor and their negative control miRNA. (**C**) HL-7702 cells were co-transfected with miR-506/miR-124 mimics and Luc-wild-type or mutant 3′-UTR of PROC plasmids. Then transcriptional activity of PROC was determined by luciferase assay. ** and ^##^Represent *P* < 0.01; ***represents *P* < 0.001; ^#^represents *P* < 0.05.

In HL-7702 and LX-2 cells, comparing with the NC group, transcriptional level of PROC was significantly lower after exposure of miR-506 and miR-124 mimics, but its level was higher after inhibiting miR-506 and miR-124 (Fig. 2A, B). Compared to the NC group, the transcriptional level of PROC was lower after exposure of miR-506 and miR-124 in HL-7702 cells transfected with wild-type PROC, but there was not significantly difference in HL-7702 cells transfected with mutated PROC (Fig. 2C). These results showed that PROC may be a target regulation gene of miR-506 and miR-124.

### miR-506 and miR-124 regulate the proliferation and apoptosis of liver cells

**Figure 3:**
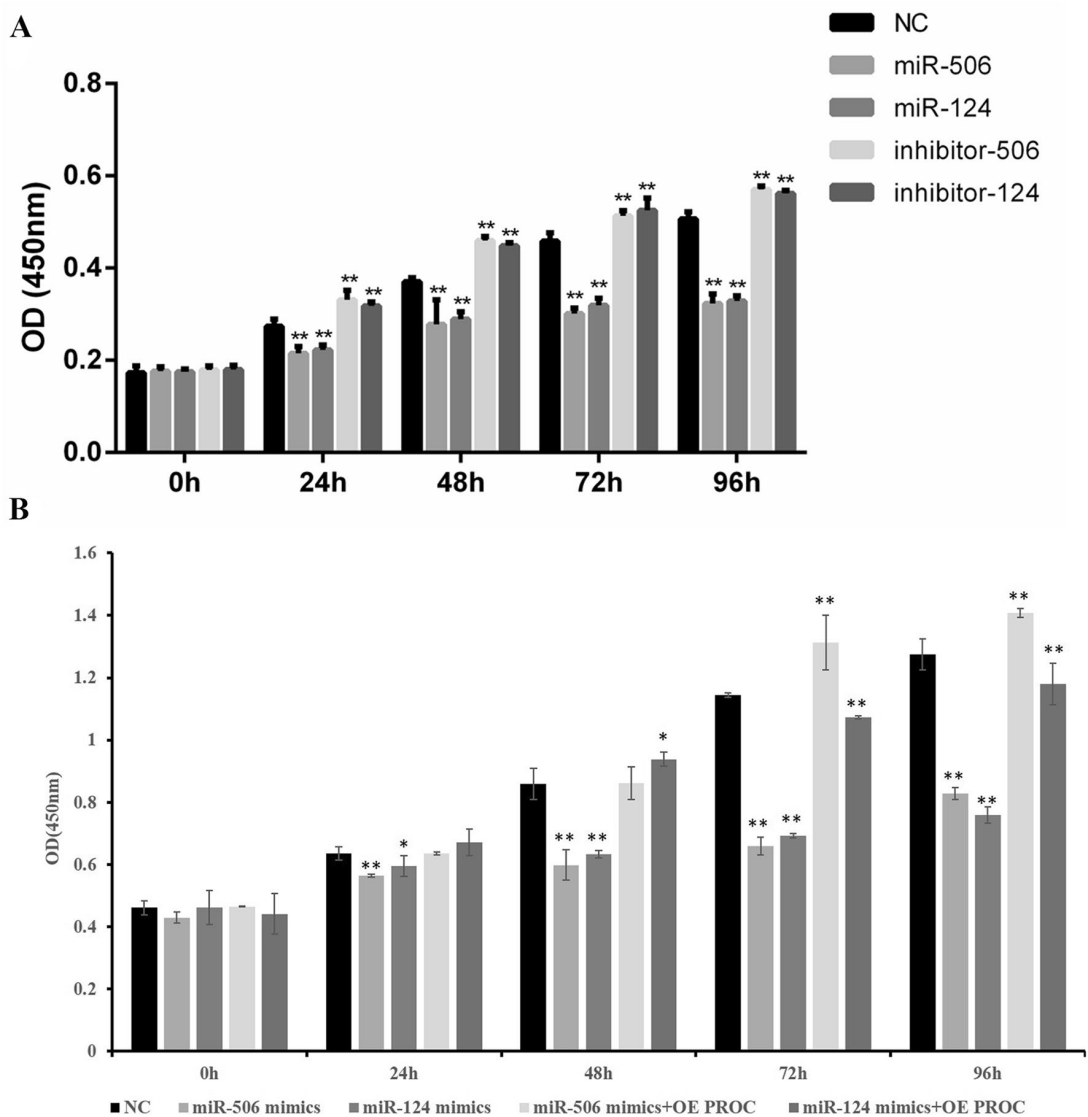
Effects of miR-506 and miR-124 on cell proliferation of liver cells. (**A**) HL-7702 cells were transfected with miR-506/124 mimics, miR-506/124 inhibitor, and negative control miRNA, and cell proliferation at 0 h, 24 h, 48 h, 72 h and 96 h was determined using CCK-8 assay. (**B**) LX-2 cells were transfected with miR-506/124 mimics, miR-506/124 mimics with overexpressing PROC, and negative control miRNA, and cell proliferation at 0 h, 24 h, 48 h, 72 h and 96 h was determined using CCK-8 assay. *OE* overexpression. **Represents *P* < 0.01, *represents *P* < 0.05.

There was no significant difference in HL-7702 cells’ proliferation among each group at 0 h. After 24 h, 48 h, 72 h and 96 h incubation, the cells’ viability was obviously lower after exposure of miR-506 and miR-124 than that in the NC group. However, the cells’ proliferation was significantly increased after inhibiting miR-506 and miR-124 at 24 h, 48 h, 72 h and 96 h (Fig. 3A). Meanwhile, the proliferation viability of LX-2 cells was lower after exposure of miR-506 and miR-124 mimics than that in the NC group. But the proliferation viability of LX-2 cells was significantly increased through overexpressing PROC (Fig. 3B).

**Figure 4:**
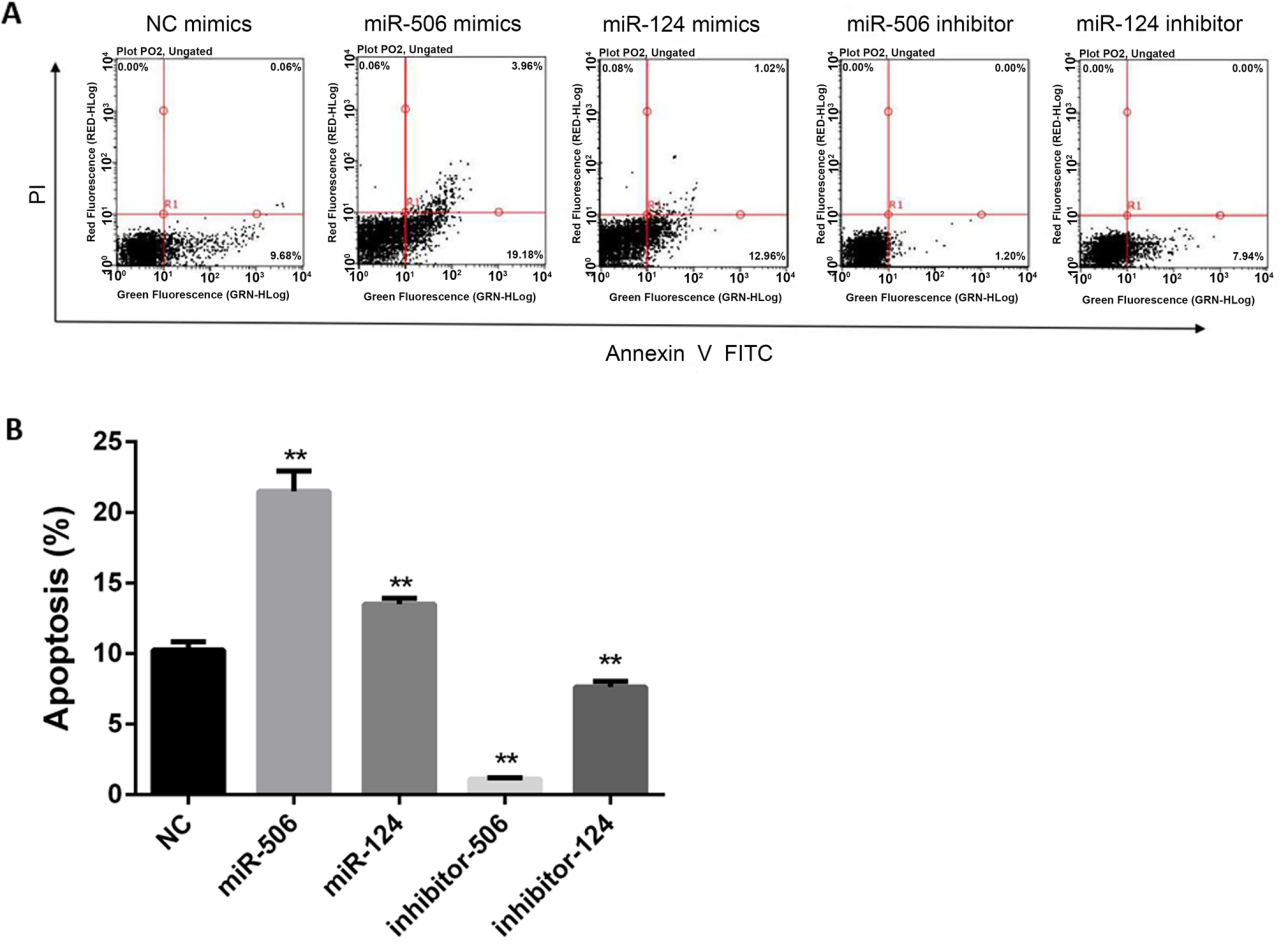
Role of miR-506 and miR-124 on apoptosis of HL-7702 cells. (**A**) Flow diagram of apoptosis of HL-7702 cells transfected with miR-506 or miR-124 mimics, inhibitor and their negative control miRNA. (**B**) The rate of apoptotic cells among different groups. **Represents *P* < 0.01.

**Figure 5:**
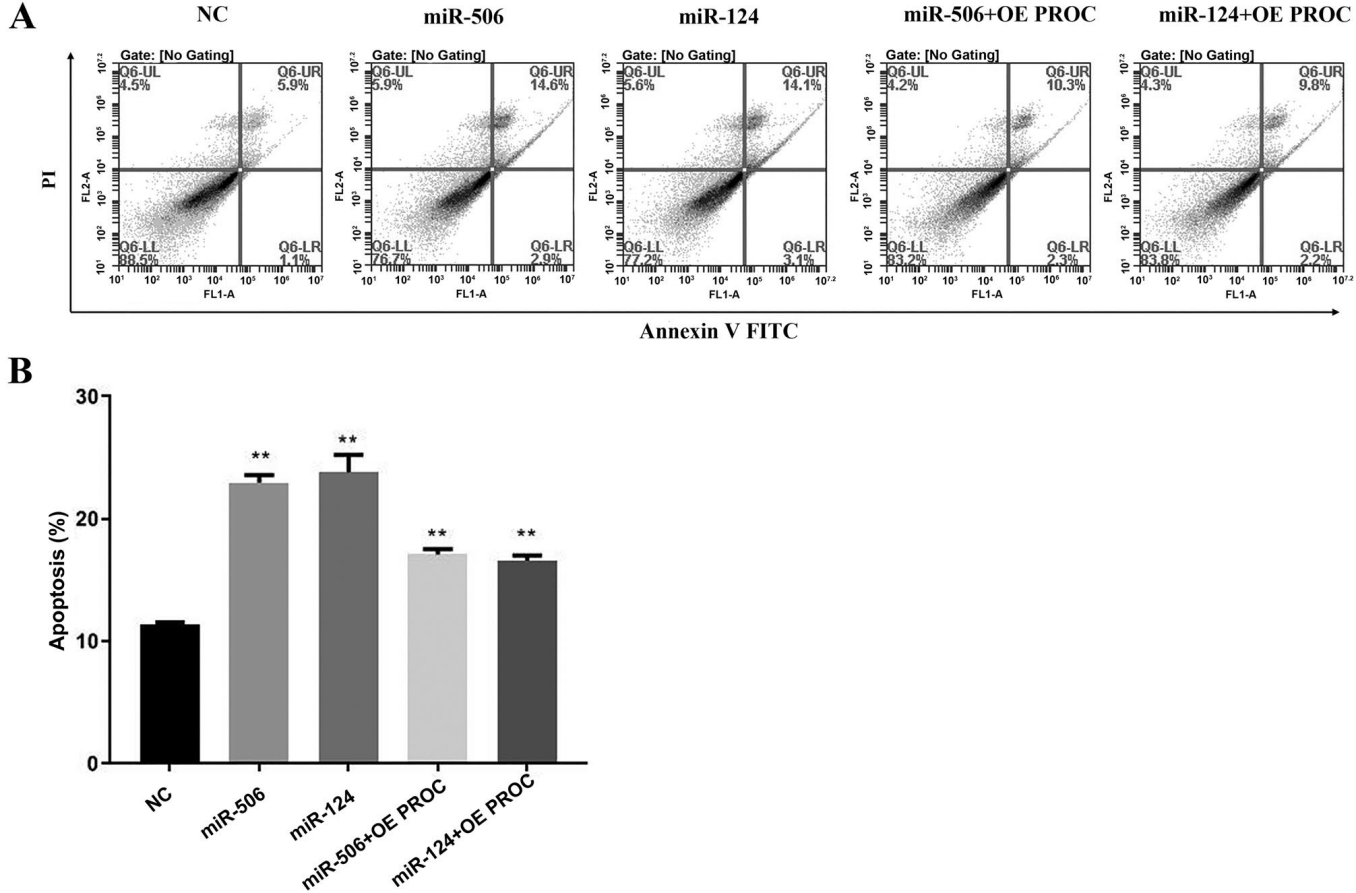
Role of PROC on miR-506 and miR-124-induced apoptosis of LX-2 cells. (**A**) Flow diagram of apoptosis of LX-2 cells transfected with miR-506 and miR-124 mimics alone, or with overexpressed PROC and their negative control miRNA. (**B**) The rate of apoptotic cells among different groups. *OE* overexpression. **Represents *P* < 0.01.

Compared with the NC group, the rate of apoptosis was significantly increased after exposure of miR-506 mimics and miR-124 mimics, which was reduced after inhibiting miR-506 and miR-124 in HL-7702 cells (Fig. 4). Furthermore, the pro-apoptosis effect of miR-506 was higher than that of miR-124. To identify the relationship between miRNA and PROC in regulation of cells’ apoptosis, a overexpressing PROC was used in LX-2 cells. The rate of apoptosis was increased by 2.5 times after exposure of miR-506 and miR-124 mimics than that in NC group, which was significantly decreased through overexpressing PROC (Fig. 5).

Based on the above results, we can found that miR-506 and miR-124 were related with the proliferation and apoptosis of liver cells, and PROC could inhibit the anti-proliferation and pro-apoptosis effect of miR-506 and miR-124.

**Figure 6:**
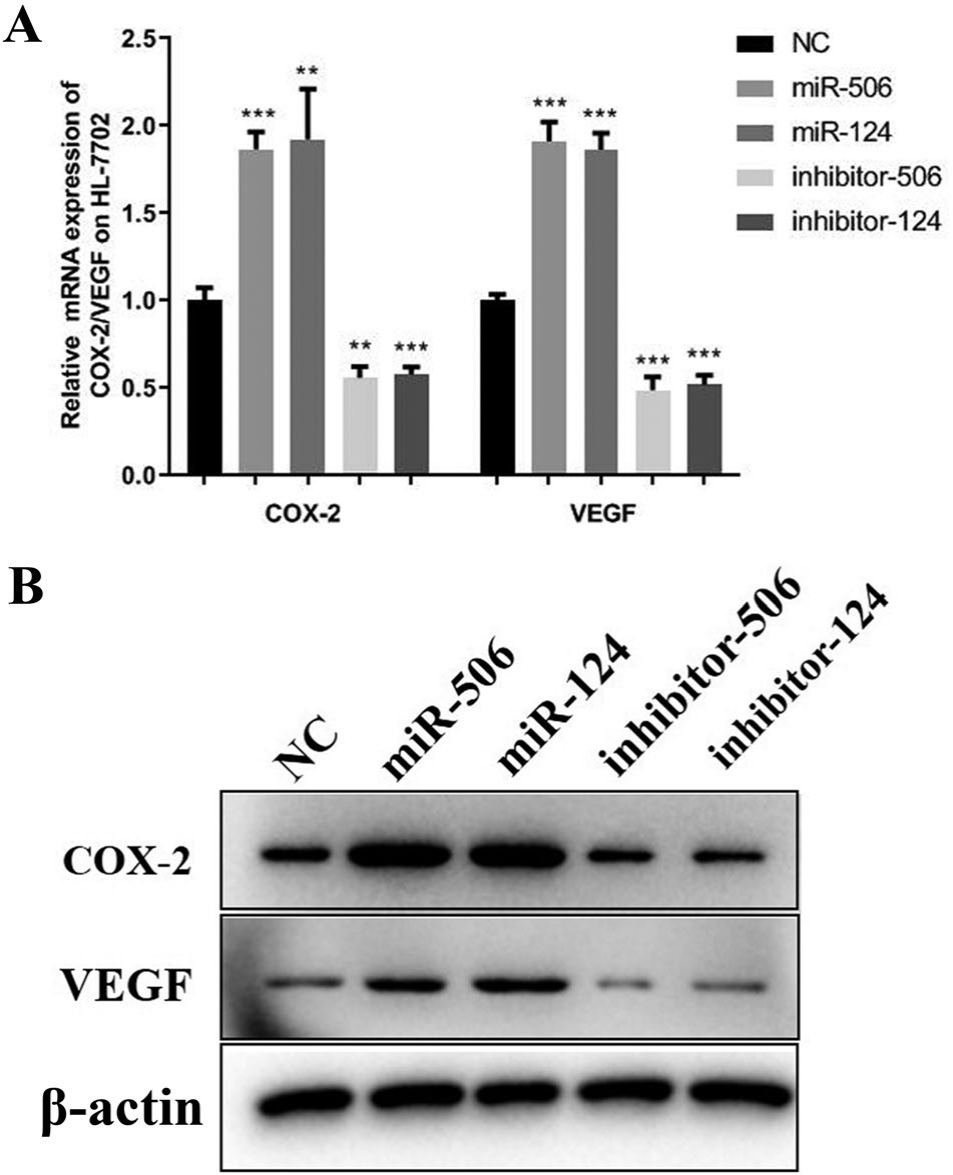
Detection of COX-2/VEGF regulated by miR-124/506. (**A**) The mRNA expression of COX-2 and VEGF was detected by qRT-PCR in HL-7702 cells. (**B**) Western Blot showed the Cox-2 and VEGF protein expression in HL-7702 cells after exposure of miR-124/506 mimics or inhibitor. **Represents *P* < 0.01, ***represents *P* < 0.001.

To identify the potential mechanism that miR-506 and miR-124 involve in the decline of PROC, some cell factors were detected. The mRNA levels of COX-2 and VEGF were significantly higher after exposure of miR-506 and miR-124 mimics than those in NC group, and this phenomenon was reversed through inhibiting miR-506 and miR-124 (Fig. 6A). Compared with the NC group, the protein levels of COX-2 and VEGF were higher after exposure of miR-506 mimics and miR-124 mimics, which was also reversed after inhibiting miR-506 and miR-124 (Fig. 6B). Furthermore, compared with the NC group, the protein levels of Bcl-2 and PROC were down-regulated and the Bax level was up-regulated after exposure of miR-506 mimics and miR-124 mimics in the Western-Blotting test. However, Bcl-2 and PROC protein levels were increased and Bax protein level was decreased after inhibiting miR-506 and miR-124.

## Discussion

At present, the most of reports in PROC deficiency focus on the family inheritance, there was only a few studies about the association between transcriptional regulation of PROC and PROC deficiency. The serum PROC is reduced in children with EHPVO due to reduced portal blood flow, and the status of reduced PROC can be improved after Rex shunt^4^. However, the level of serum PROC was still lower than normal children in 6 children with EHPVO after Rex shunt. The hereditary deficiency of PROC has been reported in portal vein thrombosis^12^, which is an autosomal genetic disease and a high risk factor for venous thrombosis. But in this study, there was no family history of DVT in the 6 cases with EHPVO, and their parents had normal level of PROC. Therefore, the causes leading to the decline of PROC are not clear in this 6 cases with EHPVO.

The single nucleotide polymorphisms (SNP) (-1654C/T, -1641A/G and -1476A/T) of PROC is associated with susceptibility to pulmonary thromboembolism in Chinese Population^13^. The thrombosis symptom is associated with the two missense mutations p. Asp297His and p. Val420Leu in the PROC gene^14^. These reports indicate PROC gene polymorphisms may involve in some thrombotic diseases. In this study, the transcriptional level of PROC was lower in sick group than that in the healthy group, which suggested that the reduced expression of PROC mRNA might involve in the occurrence and development of EHPVO. However, it is not clear that the reduction of transcriptional level of PROC is a primary or secondary event in these patients. In this study, the expression of PROC mRNA was negatively correlated with the levels of miR-506-3p and miR-124-3p, which implied the decline of serum PROC may be a secondary event regulated by miR-506-3p and miR-124-3p.

A large number of serum miRNA profiles have marked changes in patients with venous thromboembolism (VTE)^15,16^. The levels of serum miR-582, miR-195, and miR-532 are higher in patients with DVT than those in control population^17^. In addition, miR-532^18^ and miR-320a/b^19^ have been found to be up-regulated in DVT patients, which are the potential biomarkers for improving the diagnostic accuracy of DVT. Some serum miRNAs have been considered as the potential biomarkers for the diagnosis of VTE^17,20–23^. In this study, the levels of miR-124-3p and miR-506-3p were significantly higher in sick group than those in the healthy group, which were related to the decline of PROC protein and PROC mRNA. This finding suggested that miR-124-3p and miR-506-3p might be the potential biomarkers for the decline of PROC in children with EHPVO.

MiR-124-3p reduces angiotensin II (ATII)-dependent hypertension and apoptosis by down-regulating early growth response factor 1 (EGR1)^24^. MiR-506 participates in angiogenesis, which is associated with decreased expression of matrix metalloproteinase-9^25^. In the present study, the proliferation rate of liver cells was significantly decreased after exposure of miR-506 and miR-124, and this phenomenon could be reversed through inhibiting miR-506 and miR-124, which suggested miR-506 and miR-124 may involve in the proliferation of liver cells. As an endogenous anticoagulant, PROC has the potential function of promoting the cell survival^26^. In this study, the cells’ viability was lower after exposure of miR-506 and miR-124 than NC group, and the cells’ viability was increased through overexpressing PROC, which suggested that PROC may promote the liver cells’ survival. Therefore, the inhibiting role of miR-506 and miR-124 for cell proliferation may work through regulating PROC. In addition, due to PROC is synthesized in liver, the reduction of hepatocyte proliferation caused by miR-506 and miR-124 may in turn lead to the reduction of PROC secretion. Furthermore, the pro-apoptosis role of miR-506 and miR-124 in liver cells may also involve in the mechanism of regulating PROC, and the pro-apoptosis role may work through regulating PROC.

Apoptosis-related protein families can be divided into pro- and anti- apoptotic factors. The pro-apoptotic factors include Bax, Bad and Bid, and the anti-apoptotic factors have Bcl-2 and Bcl-x, which play an important role of regulating apoptosis. The interaction between Bcl-2 and Bax protein can form a heterodimer, which inhibits the apoptosis. However, a homodimer can be produced by the interaction between two Bax proteins, which promotes the apoptosis^27^. The over-expression of Bax leads to cell apoptosis by increasing the activated number of Cysteinyl aspartate- specific proteases (caspase) and antagonizing the protective effect of Bcl-2^28^. In this study, the protein level of Bcl-2 was down-regulated and the Bax level was up-regulated after exposure of miR-506 and miR-124, which may explain the potential mechanism involving in the pro-apoptosis role of miR-506-3p and miR-124-3p. But the detailed process of this mechanism is still unclear, and a further study may be needed.

Cox-2-induced vasoactive factors, including prostaglandin (PG) E_2_, thromboxane (TXA_2_) and PGI_2_, can affect the hepatic microcirculation and systemic circulation, which contribute to portal hypertension^29^. The non-selective and selective COX-2 inhibitors can reduce portal pressure in cirrhotic rats and portal hypertension model^29,30^. VEGF participates in the formation of vascular wall structure and acts as an important regulator in vasculogenesis and angiogenesis^31^. Furthermore, the vasculogenesis and angiogenesis involve in the development of lateral branches in portal venous system around portal vein, esophagus and stomach in EHPVO. In this study, the mRNA and protein levels of COX-2 and VEGF were significantly higher after exposure of miR-506 and miR-124 than the NC group, which indicated that miR-506-3p and miR-124-3p may involve in the process of portal hypertension through regulating these factors. But the mechanism is still unclear, which needs a further study.

## Conclusion

MiR-506-3p and miR-124-3p may involve in the decline of PROC in protein and transcriptional level, in which the anti-proliferation and pro-apoptosis role of miR-506-3p and miR-124-3p for liver cells may involve in this mechanism. Furthermore, the cell factors (Bax, Bcl-2, COX-2 and VEGF) mediated by miR-506-3p and miR-124-3p may also involve in the pro-apoptosis role and development of portal hypertension.

## Supplementary Information

Supplementary Information.

## Data Availability

The datasets used and/or analysed during the current study are available from the corresponding author on reasonable request.

## Abbreviations

PROC: Protein C
EHPVO: Extra-hepatic portal venous obstruction
DVT: Deep vein thrombosis
NMD: Nonsense-mediated mRNA decay
CT: Computerized tomography
COX-2: Cyclooxygenase-2
VEGF: Vascular endothelial-derived growth factor
Bcl-2: B cell lymphoma-2
BAX: BCL-2 associated X
SNP: Single nucleotide polymorphisms
VTE: Venous thromboembolism
EGR1: Early growth response factor 1
PG: Prostaglandin
TXA2: Thromboxane
NO: Nitric oxide
