# Supplementary material for: MiR-124 and miR-506 are involved in the decline of protein C in children with extra-hepatic portal vein obstruction
Source: Sci Rep. 2021 Jun 10;11:12320. doi: 10.1038/s41598-021-91862-4 (PMC8192904; doi:10.1038/s41598-021-91862-4)
Supplement: Supplementary file 1 — Supplementary Information. [file 41598_2021_91862_MOESM1_ESM.docx]

**MiR-124 and miR-506 are involved in the decline of protein C in children with extra-hepatic portal vein obstruction**

Jin-Shan Zhang^1^*, Long Li^1^

^1^Department of General Surgery, Capital institute of Pediatrics, Beijing, P.R. China

* Correspondence to:

Dr. Jinshan Zhang, Department of General Surgery, Capital institute of Pediatrics, Beijing. No.2 Yabaolu Rd. Beijing 100020, China. Telephone: 86-10-85695666, Facsimile: 86-10-85628367; E-mail: zjs851@163.com


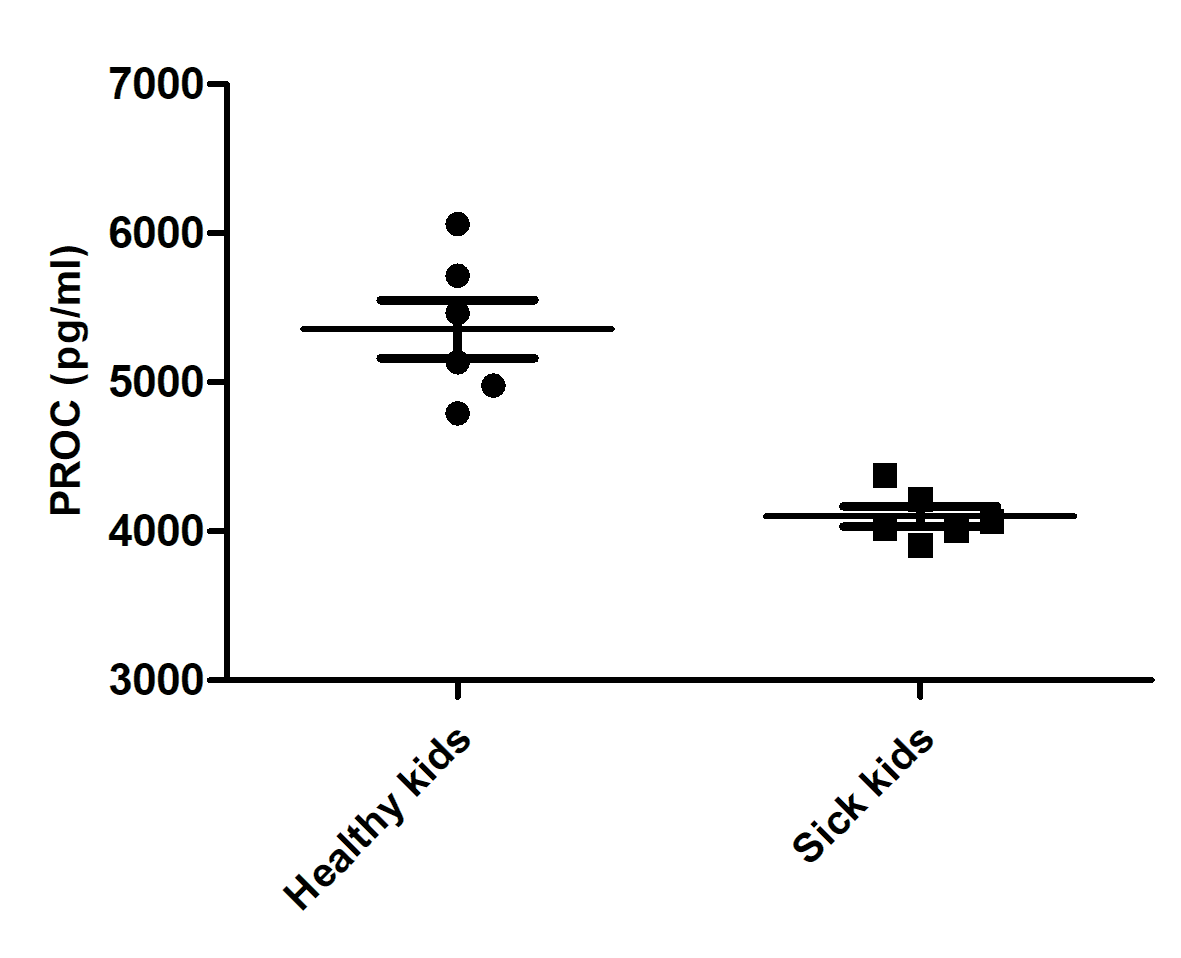


Figure 1 Scatter diagram showing the protein level of PROC detected by ELISA in Healthy Kids and Sick Kids.


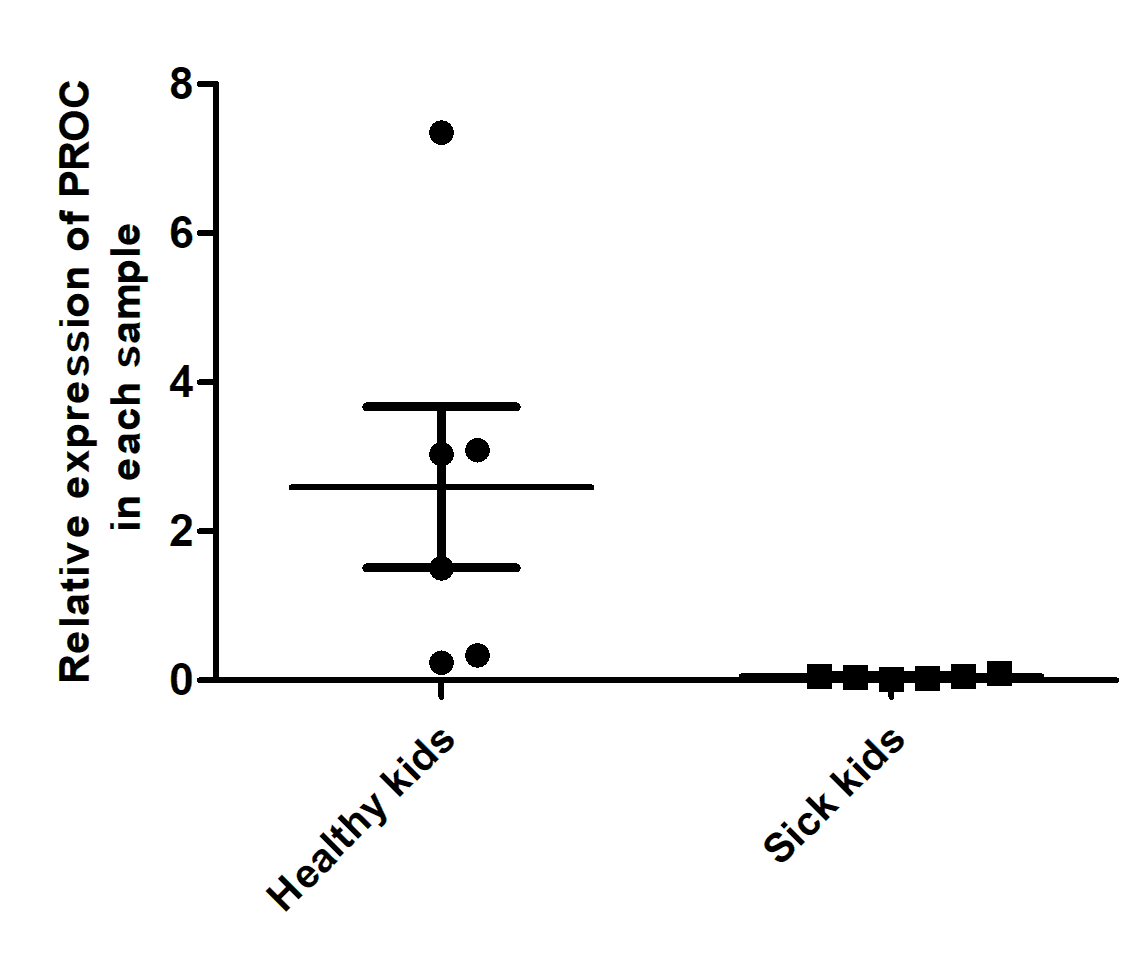


Figure 2 Scatter diagram showing the transcriptional levels of PROC detected by qPCR in Healthy Kids and Sick Kids.





Figure 3. The expressions of miR-204-5p was detected by qRT-PCR in Healthy Kids and Sick Kids.





Figure 4. The expressions of miR-211-5p was detected by qRT-PCR in Healthy Kids and Sick Kids.





Figure 5. The expressions of miR-218-5p was detected by qRT-PCR in Healthy Kids and Sick Kids.





Figure 6. The expressions of miR-802 was detected by qRT-PCR in Healthy Kids and Sick Kids.


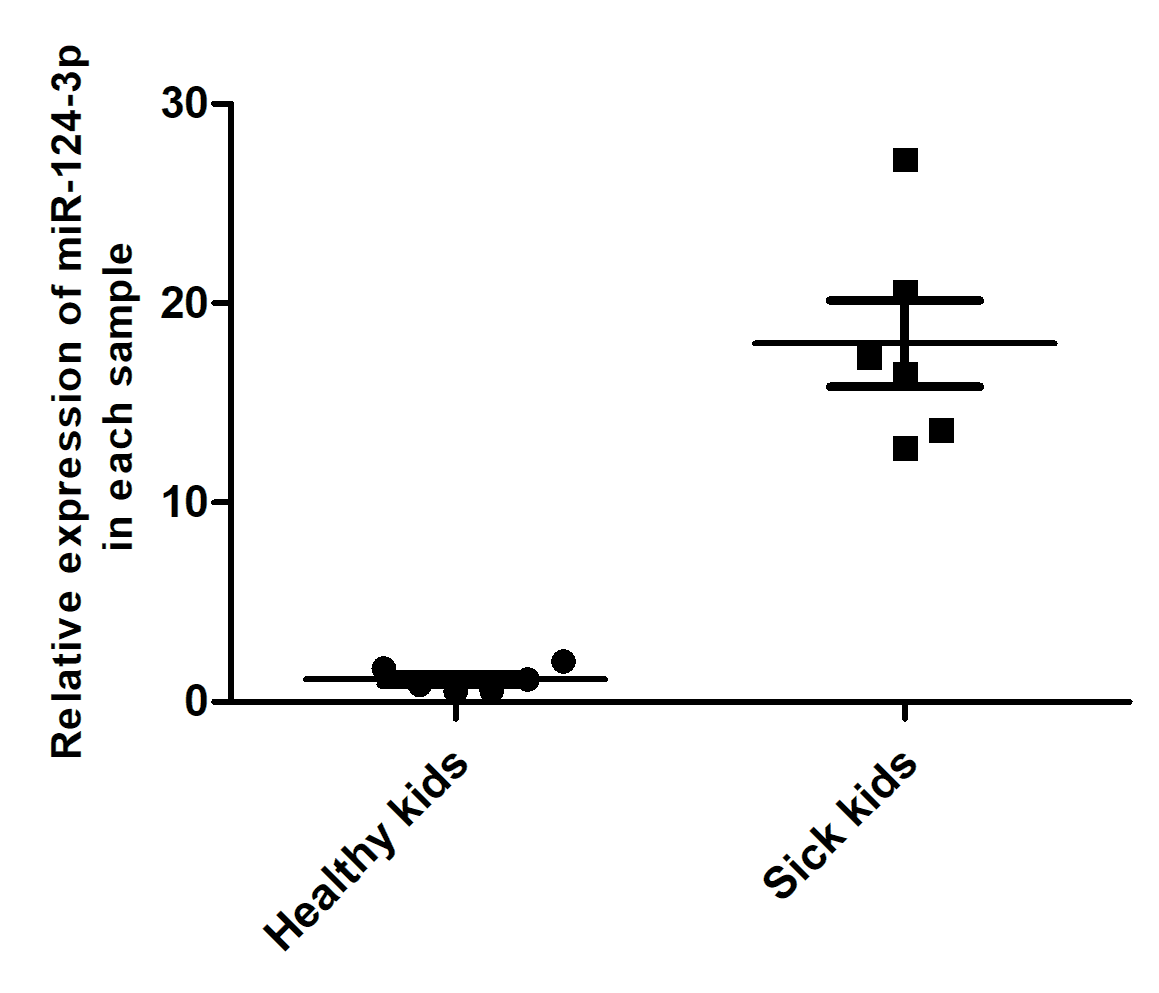


Figure 7. Scatter diagram showing the expression of miR-124-3p detected by qPCR in Healthy Kids and Sick Kids.


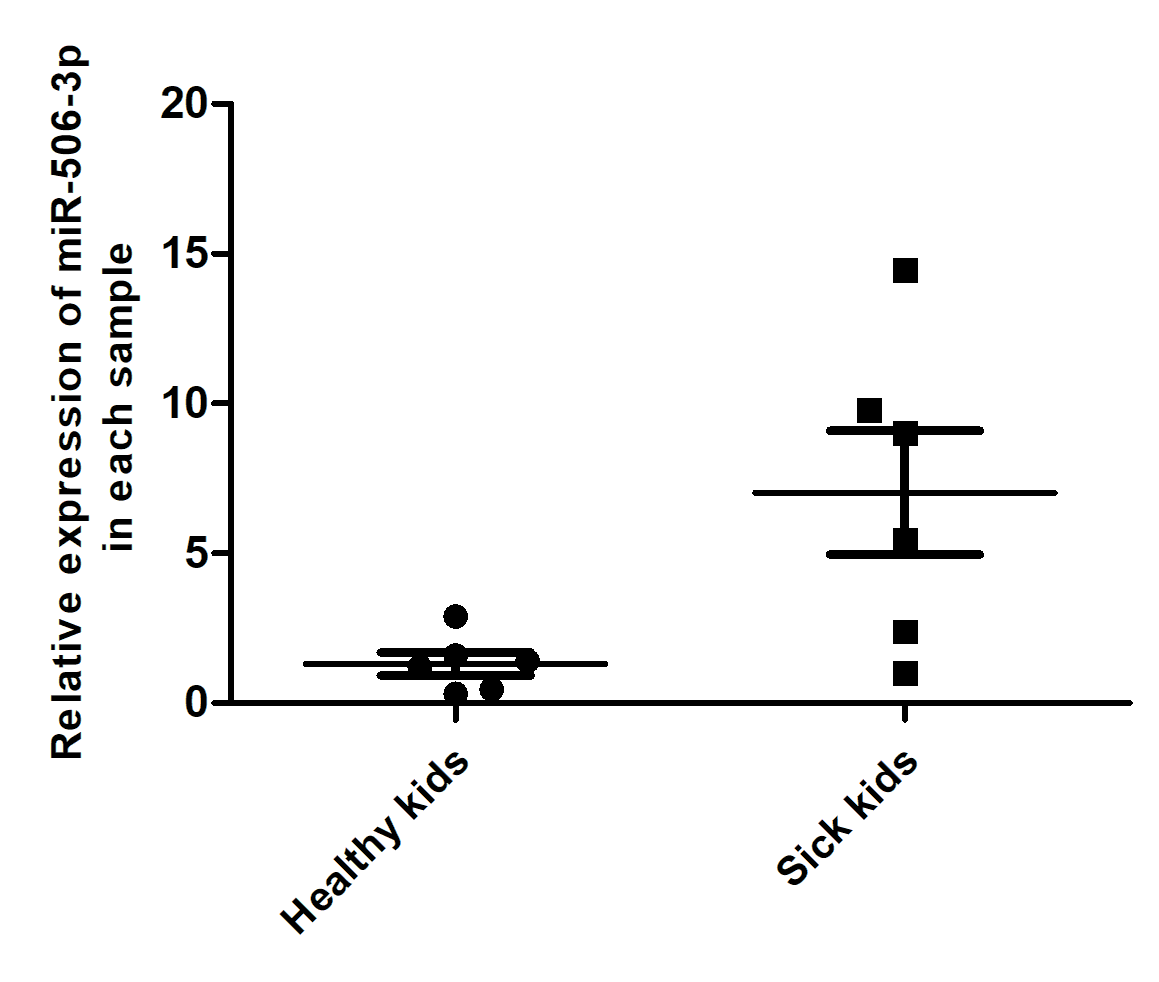


Figure 8. Scatter diagram showing the expression of miR-506-3p detected by qPCR in Healthy Kids and Sick Kids.
